# Factors Influencing Unit-Level Differences in Prevalence of Prematurity-Associated Bronchopulmonary Dysplasia in a European Cohort: An Observational Study

**DOI:** 10.1016/j.chest.2025.11.046

**Published:** 2025-12-12

**Authors:** Birte Staude, Héloïse Torchin, Rolf F. Maier, Alan C. Fenton, Pierre-Henri Jarreau, Jan Mazela, Jennifer Zeitlin, Harald Ehrhardt, EPICE-SHIPS Research Group, J. Lebeer, P. Van Reempts, E. Bruneel, E. Cloet, A. Oostra, I. Sarrechia, K. Boerch, L. Huusom, P. Pedersen, T. Weber, A. Hasselager, L. Toome, H. Varendi, M. Männamaa, P.Y. Ancel, A. Burguet, P.H. Jarreau, V. Pierrat, P. Truffert, R.F. Maier, M. Zemlin, B. Misselwitz, S. Schmidt, L. Wohlers, M. Cuttini, D. Di Lallo, G. Ancora, D. Baronciani, V. Carnielli, I. Croci, G. Faldella, F. Ferrari, F. Franco, G. Gargano, C. Koopman-Esseboom, J. Gadzinowski, J. Mazela, A. Montgomery, T. Pikuła, H. Barros, R. Costa, L. Mendes Graça, M. do Céu Machado, C. Rodrigues, T. Rodrigues, U. Aden, A.K. Edstedt Bonamy, M. Norman, E.S. Draper, E.M. Boyle, A. Fenton, S.J. Johnson, B.N. Manktelow, D.W.A. Milligan, S. Mader, N. Thiele, J. Walz, S. Petrou, S. Kim, J. Zeitlin, A.M. Aubert, M. Bonet, C. Bonnet, R. El Raffei, A. Piedvache, A.V. Seppanen

**Affiliations:** a Université Paris Cité and Université Sorbonne Paris Nord, Inserm, INRAE, Centre for Research in Epidemiology and Statistics (Obstetrical, Perinatal and Pediatric Lifecourse Epidemiology [OPPaLE]), Paris, France; b Division of Neonatology and Pediatric Intensive Care Medicine, Department of Pediatrics and Adolescent Medicine, University Medical Center Ulm, Ulm, Germany; c Department of Neonatal Medicine of Port-Royal, Cochin Hospital, FHU Prem’IMPACT, AP-HP Centre – Université Paris Cité, Paris, France; d Children’s Hospital, Philipps University of Marburg, Marburg, Germany; e Newcastle Neonatal Service, Newcastle University, Newcastle upon Tyne, England; f Department of Neonatology, Poznań University of Medical Sciences, Poznań, Poland

**Keywords:** bronchopulmonary dysplasia, chronic lung disease, cohort study, mechanical ventilation, mortality, oxygen saturation target, postnatal steroids, practice variations, very preterm

## Abstract

**Background:**

Bronchopulmonary dysplasia (BPD) is the most common morbidity of very preterm (VPT) infants born < 32 weeks’ gestation with lifelong consequences. Studies document wide variation between regions and units in BPD prevalence.

**Research Question:**

Which unit-level factors contribute to the variation in BPD prevalence among VPT infants in European neonatal units?

**Study Design and Methods:**

Analyses were conducted using the prospective population-based Effective Perinatal Intensive Care in Europe (EPICE) cohort in 19 regions in 11 European countries. We compared prevalence of moderate/severe BPD among VPT infants without severe congenital anomalies in neonatal units with ≥ 40 annual VPT admissions (83 units and 5,285 infants). Unit prevalence was adjusted for individual risk factors using standardized morbidity rates. Spearman correlation and multilevel logistic regression were used to assess associations of BPD with unit-level variables: unit mortality rates, first week oxygen saturation targets, proportion of infants ventilated within the first 24 hours, unit practice of postnatal corticosteroid use for hypotension or BPD prevention, and unit volume.

**Results:**

Unadjusted BPD prevalence ranged from 2% to 47% (median, 13%) between units and was 8% to 42% (median, 17%) after adjustment and standardization. Oxygen saturation targets, proportion of initial mechanical ventilation, and postnatal corticosteroid use partly explained the between-unit variability (proportional change of variance: 25%, 5%, and 17%, respectively), leaving 53% unexplained. Risk-adjusted in-hospital mortality (range, 8%-21%) and patient volume were not correlated with BPD prevalence.

**Interpretation:**

Our results show that large variability in BPD prevalence exists between European units, which was only partially explained by patient characteristics. Our findings suggest that improving respiratory management for VPT infants could be beneficial for reducing BPD prevalence. The association of unit postnatal corticosteroid use practice with BPD requires further investigation.

FOR EDITORIAL COMMENT, SEE PAGE 1169

**Take-Home Points**

**Research Question:** Which unit-level factors contribute to the variation in bronchopulmonary dysplasia (BPD) prevalence among very preterm infants in European neonatal units?

**Results:** Almost one-half (47%) of the variability in BPD prevalence between European neonatal units (8%-42% after case-mix adjustment) could be explained by 3 unit management factors: the unit’s first week oxygen saturation target, the proportion of infants on mechanical ventilation during the first 24 hours of life and the unit’s declared policy of postnatal corticosteroid use for arterial hypotension and BPD prevention.

**Interpretation:** The large variations in BPD prevalence across European neonatal units may be partly explained by variations in the infants’ postnatal management and indicate that BPD is not inevitable; clinicians should be encouraged to make every effort to reduce the risk of BPD.

Bronchopulmonary dysplasia (BPD), the chronic lung disease arising from preterm delivery, is the main driver of morbidity in very preterm (VPT) (< 32 weeks’ gestation) infants with long-term consequences for lung function, neurodevelopment, and somatic growth.^1^, ^2^, ^3^ Despite many advances in care and efforts to prevent BPD, its prevalence has not changed over past decades.^4^^,^^5^ Nonetheless, recent systematic reviews show wide heterogeneity in BPD prevalence between regions and units, with reported differences of up to 10-fold.^6^^,^^7^

Multiple hypotheses exist for these regional differences in BPD prevalence, but the validity and relative contributions to the observed variation is unknown. First, patient-level factors, including gestational age, birthweight, intrauterine growth restriction, male sex, and infection-driven inflammation, are well-known risks for BPD.^8^ Differences in BPD prevalence are commonly assumed to be due to variation in the distribution of these risk factors.^8^ Another hypothesized contributor is VPT mortality, which varies substantially between units and regions.^9^^,^^10^ Selective mortality of highest-risk infants, due to a less survival-focused treatment approach or linked to the organization or quality of care leading to lower survival of infants at risk for BPD, could therefore explain lower BPD prevalence.^8^ Finally, and of most interest for developing strategies for prevention, treatment approaches for VPT, differing widely between regions and units, could affect BPD prevalence.^11^ Studies linking BPD prevalence to oxygen saturation targets, ventilatory strategies, postnatal corticosteroid use, and nutritional and fluid management suggest room for improvement in unit practices, whereas those showing better outcomes with increasing unit volume, usually measured by number of admissions, and considered a marker for experience and quality of care, point to the effect of structural factors.^12^, ^13^, ^14^, ^15^

The primary aim of this study was to explore unit-level factors contributing to the variability in BPD prevalence between neonatal units in a European multicountry cohort using a standardized definition of BPD to identify factors with potential for BPD prevention.

## Study Design and Methods

### Data Source

The Effective Perinatal Intensive Care in Europe (EPICE) cohort is a prospective population-based cohort including all stillbirths and live births of 22 + 0 to 31 + 6 weeks’ gestation from 19 organizationally diverse regions in 11 European countries. Countries (regions) were Belgium (Flanders), Denmark (Eastern), Estonia (entire country), France (Burgundy, Île-de-France, and Northern), Germany (Hesse and Saarland), Italy (Emilia-Romagna, Lazio, and Marche), The Netherlands (Central and Eastern), Poland (Wielkopolska), Portugal (Lisbon and Northern), Sweden (Stockholm), and the United Kingdom (East Midlands, Northern, Yorkshire, and Humber). Data collection occurred over a 1-year period in 2011 to 2012 except for France where data were collected for 6 months. Patient data were abstracted from obstetrical and neonatal records using a standardized, pretested questionnaire. The cohort included 10,329 births, of which 7,900 were live-born. Neonatal unit questionnaires on protocols and practices were sent to units with at least 10 VPT admissions and are available for 137 neonatal units. A detailed cohort description was previously published.^16^

Ethics approval was obtained from local or hospital ethics boards, and the study was approved by the French Advisory Committee on Use of Health Data in Medical Research (CCTIRS N^o^ 13.020) and the French National Commission for Data Protection and Liberties (DR-2013-194).

### Study Population

We investigated primary neonatal units, defined as the unit where the VPT infant was hospitalized for the first consecutive 48 hours after birth. The unit level was chosen rather than a regional- or country-level approach because large variations exist in the management and outcomes within the same countries and regions. We only included units with ≥ 40 annual VPT admissions within the study period to ensure enough observations for measuring BPD prevalence and to reduce differences in case-mix. This cutoff was used instead of level of care because of the heterogeneity of classifications in Europe.^17^ One Estonian unit fulfilled inclusion criteria, but was excluded to reduce selection bias because no mechanical ventilation was provided on site. Infants with severe congenital anomalies according to the European Network of Population-Based Registries for the Epidemiological Surveillance of Congenital Anomalies (EUROCAT) definitions were excluded because they are expected to be clustered in specialized units and may have specific needs for respiratory support.^18^^,^^19^

### Definitions and Variable Selection

BPD was defined as need for supplementary oxygen or respiratory support (invasive or noninvasive mechanical ventilation or positive airway pressure) at 36 weeks’ postmenstrual age, which translates to moderate/severe BPD according to the 2001 National Institute of Child Health and Human Development consensus definition.^20^

Individual-level risk factors were chosen by clinical plausibility and literature review. They included the following: gestational age, birthweight *z* score, sex, maternal age, admission for preterm labor, maternal birth outside Europe, multiparity, and 5-minute Apgar score < 7.^8^ Birthweight *z* score was calculated using country-specific intrauterine growth references.^21^ Additionally, outborn status (defined as transfer within the first 48 hours of life) and whether the mother had received any antenatal corticosteroids before delivery were included as perinatal management factors on the individual level.

Hypotheses on unit-level effects were formulated based on literature review and selected according to availability of data within the study.^8^, ^9^, ^10^^,^^12^^,^^14^^,^^15^ Unit-level mortality rates were aggregated by unit from individual patient data as number of deaths of VPT infants in the neonatal unit or associated labor ward divided by the entire population of live-born VPT infants in the unit and labor ward. To capture oxygen exposure, oxygen saturation targets within the first week of life were retrieved from the neonatal unit questionnaires. Ventilatory strategy was defined by the aggregated proportion of infants admitted to the unit receiving invasive mechanical ventilation within the first 24 hours of life, not including Intubation-SURfactant-Extubation. For postnatal corticosteroid (PNS) use, the intention was to capture early PNS use. Therefore, it was defined as answering yes (vs no or depends) to at least one of the following items of the neonatal unit questionnaire: (1) using PNS for arterial hypotension or (2) using PNS for BPD prevention. PNS use for the treatment of BPD was not included to reduce the risk of reverse causality. Unit volume was obtained from the neonatal unit questionnaire as the total number of patients (preterm or not) treated in the neonatal unit in 2011. Sections of unit questionnaires relevant to this study are provided in e-Appendix 1. Missingness of variables ranged from 0% to 8.4% (e-Appendix 2).

### Analysis Strategy

BPD prevalence by country was calculated before exclusion of infants from units with < 40 admissions. The level of care of included and excluded units was compared by histogram using local classifications. Unadjusted prevalence by unit was calculated as number of BPD cases divided by the number of VPT admissions with data on BPD. Prevalence was then stabilized and adjusted for case-mix using a mixed effects model with the aforementioned individual-level risk factors to obtain standardized morbidity rates as defined by the Centers for Medicare & Medicaid Services (CMS) (e-Appendix 2).^22^

For ecological analysis (correlations at the unit level), each independent variable was considered separately. Correlation was assessed by Spearman ρ and visualized by scatterplots for continuous variables. Categorical variables were analyzed using Wilcoxon rank sum test and visualized by boxplots. Ecological analysis was conducted on the unadjusted and CMS-adjusted standardized BPD rates. Additionally, proportions aggregated from individual patient data were adjusted and standardized by the CMS method.

Generalized linear models with a binomial distribution and logit link function were used to account for the nested structure of the data with patients being nested in neonatal units. Unit-level variables were added to the model stepwise. Computational details can be found in e-Appendix 3.

For easier interpretation of the unit-level results, average marginal ORs (AMORs), a population-average measure that can be interpreted across units, were estimated. Moreover, median ORs were estimated to describe the magnitude of the variation between units. The median OR can be interpreted as a measure of between unit differences, representing the 50th percentile of ORs comparing patients with the same characteristics between 2 units.^23^ Proportional change in variance when adding unit-level variables was calculated starting from the model including all patient-level risk factors.^23^

Sensitivity analyses were conducted for newborns with a gestational age < 28 weeks because they are at the highest risk for BPD.^6^ Furthermore, the multilevel model was run using a unit cutoff of 30 VPT admissions and after multiple imputation by chained equation of patient-level data. Missing data for unit-level variables were not imputed. Additional sensitivity analyses were run using the original groups (no, depends, and yes) for the unit question of PNS use for arterial hypotension or BPD prevention to verify that associations were not modified by dichotomization of this variable, and for the combined outcome of BPD or death to further evaluate potential trade-offs between these 2 outcomes.

Statistical analyses were conducted using R version 4.4.2 (R Foundation for Statistical Computing), packages are detailed in e-Appendix 2. Significance was accepted at a level of *P* < .05.

## Results

**Figure 1:**
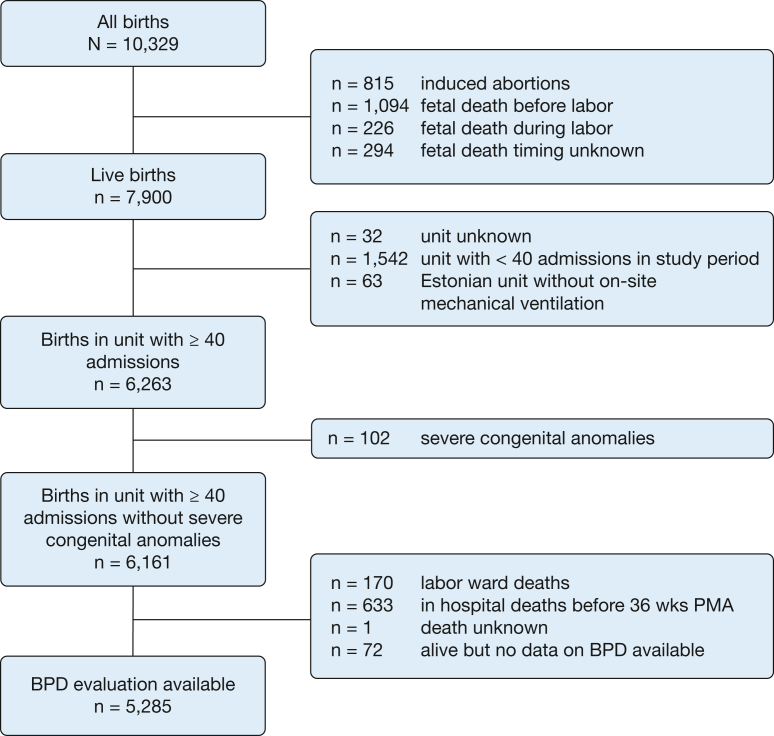
Flowchart detailing included and excluded individuals. All numbers refer to very preterm infants (< 32 wks’ gestation). BPD = bronchopulmonary dysplasia; PMA = postmenstrual age.

Of 7,900 VPT live births, 5,285 remained for analysis after applying exclusion criteria (Fig 1). Of the 242 neonatal units, 83 had ≥ 40 annual admissions, which included 81% of level 3 and 24% of level 2 units (e-Fig 1). A total of 886 infants (16.8%) fulfilled the criteria of BPD. Infants with BPD were more immature and less likely female, had lower birthweight *z* scores, and had more frequently Apgar scores < 7. Mothers were less likely to be born outside of Europe and to be admitted for premature labor (Table 1).

**Table 1:** Baseline Characteristics of Mothers and Infants Included in the Analysis, Separated by Diagnosis of BPD

Characteristic	Total	No BPD	BPD
	(N = 5,285)	(n = 4,399)	(n = 886)
Gestational age, wk	29.7 (27.7-31.0)	30.0 (28.4-31.1)	27.0 (25.7-28.6)
Birthweight, g	1,205 (945-1490)	1,280 (1,030-1,540)	835 (710-1,023)
Birthweight *z* score	−0.65 (−1.67 to 0.17)	−0.62 (−1.55 to 0.20)	−0.87 (−2.27 to 0.01)
	1 (0.0)	1 (0.0)	0 (0.0)
Female sex	2,474 (46.8)	2,109 (48.0)	365 (41.2)
	1 (0.0)	1 (0.0)	0 (0.0)
Multiple	1,697 (32.12)	1,463 (33.27)	234 (26.41)
	1 (0.0)	1 (0.0)	0 (0.0)
5-min Apgar score < 7	767 (15.3)	534 (12.7)	233 (28.4)
	259 (4.9)	194 (4.4)	65 (7.3)
Maternal age, y	31 (26-35)	31 (27-35)	30 (26-34)
	22 (0.4)	17 (0.4)	5 (0.6)
Mother born outside Europe	929 (18.2)	800 (18.8)	129 (15.1)
	168 (3.2)	138 (3.1)	30 (3.4)
Multiparous	2,275 (43.0)	1,888 (43.4)	387 (43.8)
	48 (0.9)	45 (1.0)	3 (0.3)
Admission for premature labor	2,494 (48.3)	2,083 (48.4)	411 (47.4)
	118 (2.2)	99 (2.3)	19 (2.1)
Antenatal corticosteroids (any)	4,736 (89.6)	3,946 (90.4)	790 (89.8)
	39 (0.7)	33 (0.8)	6 (0.7)
Outborn status	491 (9.3)	376 (8.6)	115 (13.0)

Data are presented as median (interquartile range) for continuous variables and count (%) for discrete values. Second rows of count (%) are indicating missingness for the respective variable. BPD = bronchopulmonary dysplasia; Outborn = transfer within the first 48 h of life.

**Figure 2:**
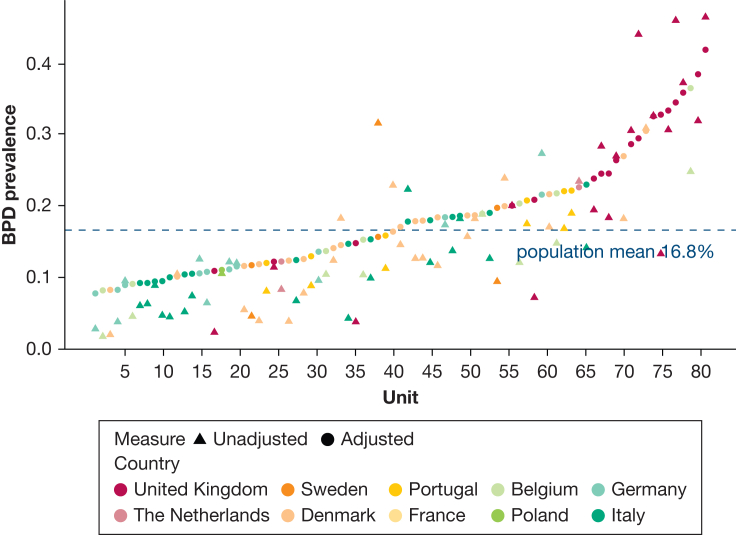
Unadjusted (triangles) and adjusted (by standardized morbidity rates, dots) prevalence of BPD by unit. BPD = bronchopulmonary dysplasia.

**Figure 3:**
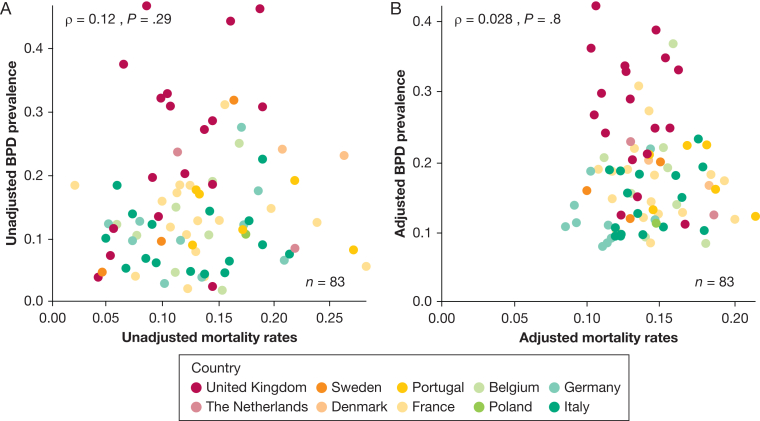
A, B, Scatterplots showing correlation of mortality and prevalence of BPD (A) for unadjusted BPD and mortality rates and (B) adjusted by standardized mortality and morbidity rates. BPD = bronchopulmonary dysplasia.

**Figure 4:**
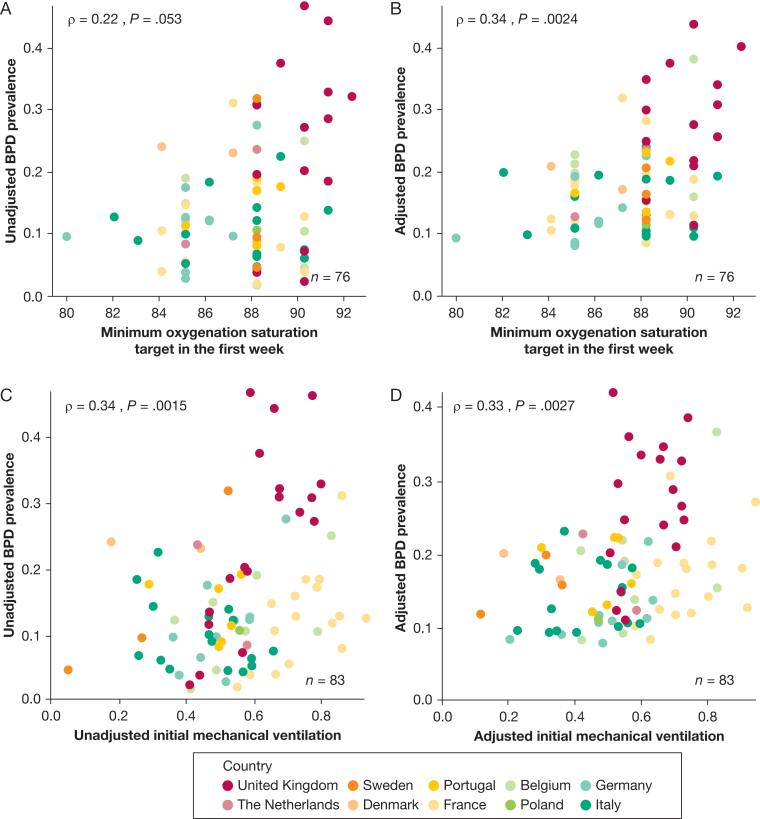
A-D, Ecological analysis of respiratory management. Correlation of minimum oxygen saturation targets in the first week of life (A, B) and of proportion of infants intubated and mechanically ventilated within the first 24 h of life (C, D) with unadjusted (A and C) and adjusted (B and D) prevalence of BPD. BPD = bronchopulmonary dysplasia.

**Figure 5:**
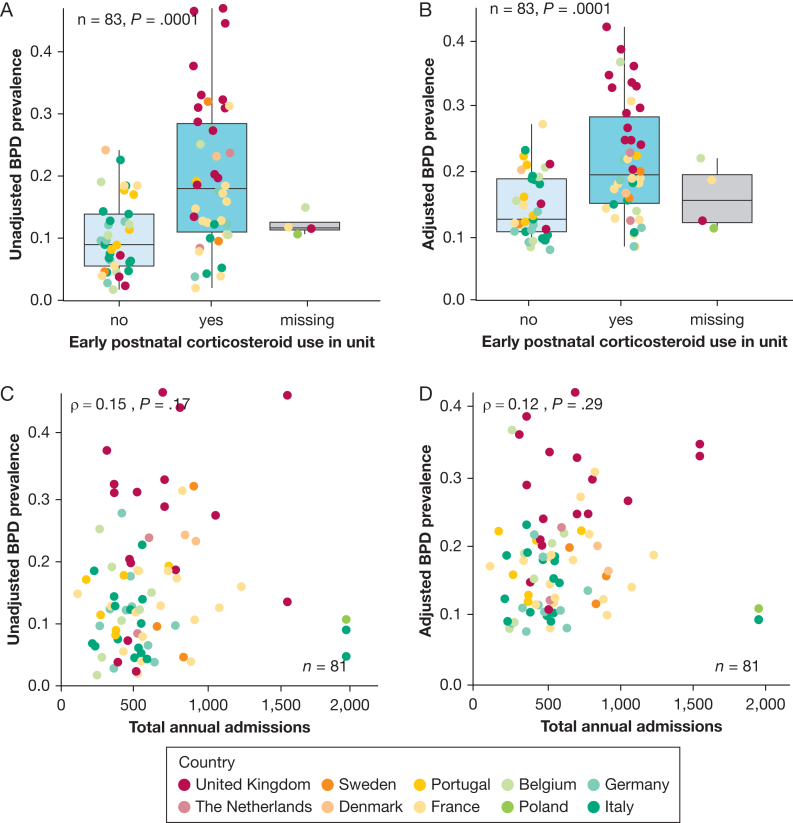
A-D, Ecological analysis of other practices and characteristics. Association of unit use of early postnatal corticosteroids for arterial hypotension or BPD prevention with unadjusted (A) and adjusted (B) prevalence of BPD. Correlation of unit volume (measured by total annual admissions) to the unit with unadjusted (C) and adjusted (D) prevalence of BPD. The 3 units with > 2,000 admissions were grouped together at 2,000. BPD = bronchopulmonary dysplasia.

Unadjusted BPD prevalence ranged from 10% in Italy to 26% in the United Kingdom (e-Fig 2) and from 2% to 47% (median, 13%) across units narrowing to 8% to 42% (median:17%) after stabilization and adjustment for case-mix (Fig 2). Ecological analysis revealed no correlation at the unit level between BPD prevalence and mortality rates before and after adjustment (Fig 3). Higher oxygen saturation targets and respiratory management with invasive mechanical ventilation in the first 24 hours showed a positive correlation with BPD prevalence (Fig 4). PNS use was reported by 42 units (52%, 4 missing, 33 for hypotension treatment, 4 for BPD prevention, and 5 for both indications). Reported PNS use was associated with higher BPD prevalence (Figs 5A, 5B). There was no association between unit volume and BPD prevalence (Figs 5C, 5D).

The AMORs are displayed in Table 2. Significant individual-level risk factors were those identified in descriptive analyses. Confirming ecological analyses, first-week oxygen saturation targets, the proportion of initial mechanical ventilation, and PNS use for hypotension or BPD prevention were significant in stepwise multilevel models, but only oxygen saturation targets and PNS use remained significant in the fully adjusted model.

**Table 2:** Multilevel Models With Average Marginal ORs for Population and Unit Factors (Added Stepwise) Presented With 95% CIs

Variable	Population	Selective Mortality	Saturation Targets	Mechanical Ventilation	Postnatal Corticosteroids	Unit Volume
	(n = 4,669; 83 Units)	(n = 4,669; 83 Units)	(n = 4,286; 76 Units)	(n = 4,286; 76 Units)	(n = 4,034; 74 Units)	(n = 4,034; 74 Units)
Gestational age, wk	0.50 (0.47-0.52)	0.49 (0.47-0.52)	0.49 (0.46-0.52)	0.49 (0.46-0.52)	0.49 (0.46-0.52)	0.49 (0.46-0.52)
Birthweight z score	0.60 (0.55-0.65)	0.60 (0.55-0.65)	0.60 (0.56-0.66)	0.60 (0.56-0.66)	0.61 (0.56-0.66)	0.61 (0.56-0.66)
Female sex	0.63 (0.52-0.77)	0.64 (0.52-0.77)	0.65 (0.53-0.80)	0.65 (0.53-0.80)	0.65 (0.53-0.81)	0.65 (0.53-0.80)
5-min Apgar score < 7	1.77 (1.39-2.24)	1.77 (1.39-2.24)	1.82 (1.42-2.33)	1.81 (1.41-2.32)	1.89 (1.47-2.44)	1.89 (1.47-2.44)
Maternal age, y	1.09 (0.92-1.30)	1.09 (0.92-1.30)	1.11 (0.93-1.33)	1.13 (0.94-1.35)	1.10 (0.92-1.33)	1.10 (0.92-1.33)
Mother born outside Europe	0.56 (0.43-0.74)	0.56 (0.43-0.74)	0.50 (0.38-0.68)	0.50 (0.37-0.67)	0.49 (0.37-0.66)	0.49 (0.37-0.66)
Multiparous	1.01 (0.83-1.24)	1.01 (0.83-1.24)	0.99 (0.80-1.23)	0.98 (0.79-1.22)	0.99 (0.79-1.23)	0.99 (0.79-1.23)
Admission for preterm labor	0.77 (0.62-0.95)	0.77 (0.62-0.95)	0.77 (0.62-0.96)	0.77 (0.62-0.96)	0.72 (0.58-0.91)	0.73 (0.58-0.91)
Antenatal corticosteroids (any)	0.78 (0.55-1.11)	0.78 (0.55-1.11)	0.80 (0.56-1.15)	0.82 (0.57-1.18)	0.81 (0.55-1.18)	0.80 (0.55-1.18)
Outborn status	1.23 (0.88-1.74)	1.23 (0.88-1.74)	1.33 (0.92-1.91)	1.30 (0.90-1.87)	1.34 (0.92-1.94)	1.34 (0.92-1.94)
Mortality, %	NA	0.99 (0.96-1.03)	1.00 (0.96-1.04)	0.99 (0.96-1.03)	1.00 (0.96-1.03)	1.00 (0.97-1.03)
Saturation targets in the first week of life, %		NA	1.18 (1.08-1.29)	1.15 (1.06-1.26)	1.15 (1.061.25)	1.15 (1.06-1.25)
Mechanical ventilation within 24 h, 10%		NA	NA	1.14 (1.01-1.28)	1.04 (0.92-1.17)	1.03 (0.92-1.17)
Early postnatal corticosteroid use		NA	NA	NA	1.99 (1.32-2.99)	2.04 (1.35-3.09)
Unit volume	NA	NA	NA	NA	NA	0.98 (0.92-1.04)
Variance	0.65	0.65	0.49	0.46	0.35	0.34
PCV, %	NA	0.09	25.13	29.82	46.83	47.35
Median OR	2.16	2.16	1.95	1.91	1.76	1.75

Data are presented as average marginal ORs (95% CI) or as otherwise indicated. PCV and median OR of each model are shown. NA = not applicable; Outborn = transfer within the first 48 h after birth; PCV = proportional change in variance.

Although the median OR decreased from 2.16 to 1.75 when adding in unit-level variables, it remained elevated, indicating remaining variance at the unit level. In the full model, proportional change in variance amounted to 47%, leaving 53% of the variance unexplained (Table 2).

**Figure 6:**
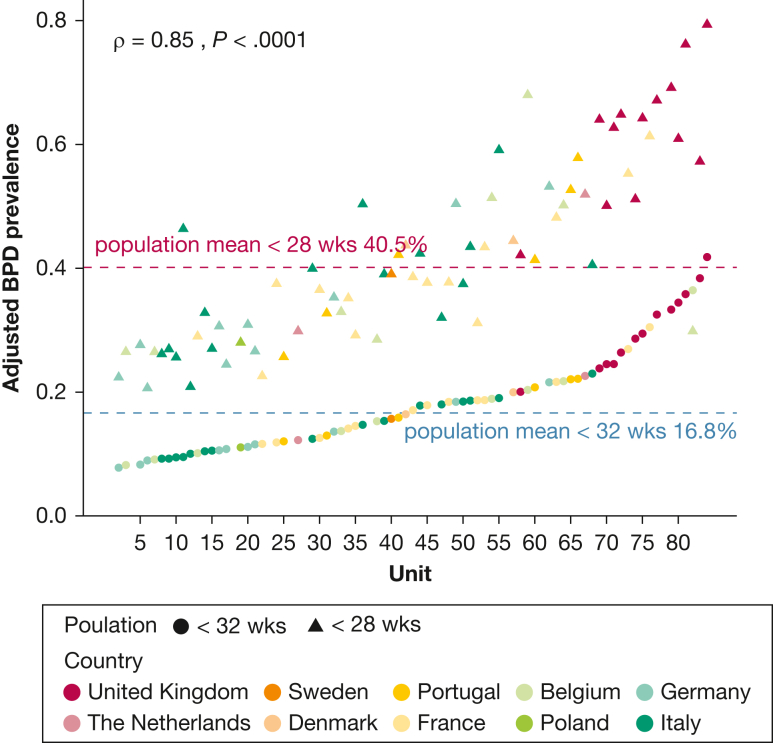
Adjusted prevalence of BPD by unit for all included infants (< 32 wks’ gestational age, dots) and infants at high risk (< 28 wks’ gestational age, triangles). BPD = bronchopulmonary dysplasia.

BPD prevalence in the population < 28 weeks of age was higher than in the whole sample (40.5%) with similar interunit variability (Fig 6) and results in multilevel models: AMOR of 1.10 for oxygen saturation targets and 2.43 for PNS use for hypotension or BPD prevention (e-Table 1). Multiple imputation of missing independent variables and inclusion of units with ≥ 30 admissions in the study period did not change the results of the multilevel model in a meaningful way (e-Tables 2, 3). A unit’s declared use of PNSs remained a risk factor, compared with no declared use, even when not including depends with the no category (e-Fig 3, e-Table 4). Using the combined outcome of BPD or death yielded similar results (e-Table 5).

## Discussion

Our study found wide heterogeneity in BPD prevalence between European neonatal units, which was only partially explained by patient-level characteristics and could not be explained by selective mortality or unit volume. However, unit-level variables explained about one-half of the variance between units, suggesting the potential for improvement by modifying practices. These variables were the following: (1) unit oxygen saturation targets in the first week of life, (2) proportion of mechanical ventilation within the first 24 hours of life, and (3) unit practice of PNS use for treatment of arterial hypotension or BPD prevention.

Our findings showing wide heterogeneity in BPD prevalence are concordant with a recent systematic review.^6^ Prevalence rates are in line with reports from neonatal networks between 2010 and 2015 (Sweden, 18%-30%; Italy [Tuscany], 7%-11%; and United Kingdom, 32%-38%).^5^ The continued relevance of our data collected in 2011 and 2012 is underlined by recent data from included countries that find comparable BPD prevalence, with 5% to 7% in Germany and 29% to 32% in the United Kingdom.^24^^,^^25^ Our results confirmed well-established patient-level risk factors, but taking these into consideration did not substantially reduce between-unit variability, as also shown by a California Perinatal Quality Care Collaborative Study.^8^ The observed wide variability between units in mortality rates, even after taking into account their patient case-mix, and their variations in practices like initial mechanical ventilation, oxygen saturation targets, and PNS use for treatment of arterial hypotension or BPD prevention, is consistent with previous reports and highlights the lack of standardized care protocols for VPT infants.^10^^,^^12^^,^^26^^,^^27^

### Strengths and Limitations

A strength of this study is the use of population-based real-life multinational data collected using the same standardized and pretested tool. This adds confidence to the measurement of crude and adjusted BPD prevalence across units. Moreover, the availability of unit questionnaire data in addition to individual patient data is a benefit. One limit is the age of the data. Changes in practice occurred since, with more active care for preterm infants at the border of viability, optimized timing of antenatal steroid application, more restrictive approaches to mechanical ventilation together with less-invasive surfactant application, and reduction of prolonged courses of antibiotic therapy.^28^, ^29^, ^30^, ^31^, ^32^ Even though BPD prevalence remains high and variability in BPD is still pronounced, validation of our findings in a more recent cohort would allow for better generalizability to current care practices.^4^ However, to our knowledge a cohort focusing on unit-level management with a similar diversity of units does not currently exist. Furthermore, it was not possible to define a unit-questionnaire variable for some of the hypotheses, making it necessary to use aggregated patient-level data. Although this is a commonly used method, we would have preferred data that was completely independent of the individual patient.^33^ Additionally, saturation targets might differ beyond the first week of life during the neonatal unit stay, and the reported unit-level practices may not always translate into individual-level care. Therefore, the unit’s saturation targets might not always correctly reflect the individual patient’s oxygen exposure. The number of executed oxygen reduction tests was low, prohibiting the use of a physiological definition of BPD.

## Interpretation

Our data suggest that unit-level respiratory management is associated with BPD. Our findings on oxygen saturation targets are in line with a meta-analysis of randomized controlled trials (RCTs) showing a reduction in odds for BPD of 19% when applying lower oxygen saturation targets (85%-89% vs 91%-95%).^34^ This meta-analysis also showed higher mortality in the lower target group, whereas our data did not support a trade-off between mortality and BPD. However, comparability of our data with these RCTs is limited because none of our units had a target range corresponding to the lower group in these RCTs with an upper limit < 90% (e-Fig 4).

Although the proportion of infants on mechanical ventilation within the first 24 hours after birth in the unit did not remain a significant risk factor for BPD in fully adjusted models, a meta-analysis has shown a reduction of 11% in odds for BPD when promoting noninvasive ventilation.^28^ Trials on initial nasal CPAP therapy vs intubation in the delivery room and less-invasive surfactant application showed a reduced risk of BPD, underlining the benefit of preventing mechanical ventilation.^28^^,^^29^ The pathomechanistic understanding of oxygen toxicity and tissue stretch (as a correlate of mechanical ventilation) causing inflammation and lung injury supports these findings on the importance of respiratory management.^35^ Furthermore, some quality improvement studies focusing on standardization of ventilatory practices, including less invasive ventilation, have shown reduced BPD prevalence.^36^ In our study, the proportion of early mechanical ventilation lost significance after including units’ reported PNS use for treatment of arterial hypotension or BPD prevention in the model, indicating that these correlated unit-level variables at least partially explain the same part of the model’s variance.

The positive association of BPD with unit PNS use is contrary to what we expected because a reduction in BPD risk with PNS use has been shown by individual patient data meta-analysis of RCTs.^37^ Our results have to be interpreted cautiously, given the risk of ecological fallacy that can occur by assuming that an association between PNS use and BPD at the unit level reflects individual risks. Furthermore, although we intentionally excluded the use of PNS for BPD treatment, we cannot completely exclude the possibility of reverse causality if units using PNS for BPD treatment are also more likely to use it for hypotension and BPD prevention.

Additionally, this variable might reflect differences in unit practices related to hypotension management or risks of hypotension not accounted for by patient-level adjustments because the variable was mainly driven by that indication in our data. However, PNS use for hypotension has mostly not been evaluated by the previous large RCTs. Finally, although PNS use may lead to shorter times of mechanical ventilation through earlier extubation, it also results in alveolar simplification in rodent models.^37^^,^^38^ Given continuing adoption of less invasive ventilatory approaches, there may be a tipping point where harmful effects on the lung tissue outweigh benefits from reducing iatrogenic harm.^39^^,^^40^ These results suggest a need to reevaluate combined PNS use for BPD prevention and treatment of arterial hypotension within current care settings while taking the multidimensional aspects of ventilatory practices like oxygen exposure, tidal volumes and airway pressure, severity of lung inflammation and gas exchange disturbance, and the timing of PNS use into account.^41^

Although we did not find an association of unit volume with BPD, this association has been demonstrated in previous studies with one suggesting that this effect is mainly seen in units with low numbers of VPT admissions.^12^^,^^42^ The lack of an association in our data might therefore be due to the exclusion of these units.

### Future Research

Only 47% of the variance in our multilevel model could be explained by unit variables, but relevant variance on the unit level remained, which could be due to patient- or unit-level factors not included in the analysis. Relevant unit practices that we were unable to measure in our study were, for example, antibiotic exposure, fluid intake, nutrition, and specifications of the ventilatory approach like tidal volumes and airway pressure and duration of mechanical ventilation.^13^^,^^31^^,^^32^^,^^35^^,^^43^, ^44^, ^45^ Additionally, oxygen saturation targets will not only influence oxygen exposure, but also weaning practices, possibly leading to differences in BPD classification, demanding future research to separate the relative impact of these factors. Furthermore, future studies might benefit from unbiased machine learning approaches for the identification of novel management factors.

In summary, we observed wide heterogeneity in BPD prevalence between European neonatal units after adjustment for patient-level risk factors which was not explained by selective mortality or unit volume. Our data suggest that a relevant proportion of this variability was due to differences in respiratory management, including mechanical ventilation for initial respiratory stabilization of infants and oxygen saturation targets in the first week of life. The interpretation of results on unit PNS use for hypotension or BPD prevention and BPD was less straightforward, raising questions orienting further investigation. In a broader perspective, this study highlights the potential for insight into the intractable problem of high and persistent BPD rates through a better understanding of care practices and their effect on BPD risks and provides starting points for hypothesis-driven research to reduce the BPD burden.

## Funding/Support

The EPICE study was funded by the 10.13039/501100000780European Union [Seventh Framework Programme FP7/2007–2013, No. 259882].

## Financial/Nonfinancial Disclosures

None declared.

## Supplementary Data

e-Online Data
