# Supplementary material for: Factors Influencing Unit-Level Differences in Prevalence of Prematurity-Associated Bronchopulmonary Dysplasia in a European Cohort: An Observational Study
Source: Chest. 2025 Dec 12;169(5):1298–310. doi: 10.1016/j.chest.2025.11.046 (PMC13197967; doi:10.1016/j.chest.2025.11.046)
Supplement: e-Online Data [file mmc1.docx]

**Online Supplement:** **Factors influencing unit-level differences in prevalence of prematurity associated bronchopulmonary dysplasia in a European cohort: an observational study**

Birte Staude, Héloïse Torchin, Rolf F. Maier, Alan C. Fenton, Pierre-Henri Jarreau, Jan Mazela, Jennifer Zeitlin, Harald Ehrhardt for the EPICE-SHIPS Research Group^7^

**Supplemental Material**

Contents:

**e-Appendix 1.**

**e-Appendix 2.**

**e-Appendix 3.**

**e-Figure 1.**

**e-Figure 2.**

**e-Figure 3.**

**e-Figure 4.**

**e-Table 1.**

**e-Table 2.**

**e-Table 3.**

**e-Table 4.**

**e-Table 5.**

**References**

**e-Appendix 1**

*Unit-level variables from the neonatal unit questionnaire*

The following questions were used to define the corresponding unit variables:

1. Minimum oxygen saturation targets in the first week of life:

In your unit, what oxygen saturation targets are used for infants less than 32 weeks GA in the first week of life? __% to __%

1. Postnatal corticosteroid use for arterial hypotension and BPD prevention:

Are systemic postnatal corticosteroids given to infants less than 32-week GA for:
(*tick all that apply*)

1. Treatment of arterial hypotension?:
   1. Yes
   2. no (or only in exceptional circumstances)
   3. no policy (decision made by the attending physician)*
2. Prevention for BPD?
   1. Yes
   2. no (or only in exceptional circumstances)
   3. no policy (decision made by the attending physician)*
3. Treatment of BPD?**
   1. Yes
   2. no (or only in exceptional circumstances)
   3. no policy (decision made by the attending physician)*

* referred to as depends
** this part of the question was not included in the analyses to avoid reverse causality

1. Annual number of admissions/ unit volume:
   Total number of admissions of babies* in 2011 from maternity units and other neonatal units: __
   (*Definition of baby is <28 days postnatal age)

**e-Appendix 2.**

*Missing data*

Few surviving infants had missing BPD status (<2%), due principally to transfers out of the regions, and were not included in the analysis. Unit-level data were missing for unit volume, saturation targets and use of postnatal corticosteroids for 2.4%, 8.4% and 4.8% respectively. Missingness for individual-level variables ranged from 0% to 4.9%.

**e-Appendix 3.**

*Adjustment and Stabilization*

BPD prevalence as well as unit mortality and proportion of initial mechanical ventilation were adjusted and stabilized using standardized morbidity and mortality rates as suggested by the Centres for Medicare & Medicaid Services (CMS)^1^. A mixed model with only level 1 variables and a random intercept by neonatal unit was fitted on the data set of all included infants using R package lme4^2^. Predicted and expected BPD prevalence by unit were obtained by estimating model predictions with and without random effects respectively. Standardized BPD rate was calculated thereof as (predicted BPD rate/expected BPD rate)*overall BPD rate.

*Multilevel models*

Multilevel models were fitted using R package lme4^2^. Models were estimated using Maximum Likelihood with Adaptive Gauss-Hermite Quadrature with 10 quadrature points and BOBYQA optimizer.

Predictors were centred to the grand mean. In addition to a linear approach, continuous variables were modelled using restricted cubic splines as well as second- and third-degree polynomials. As Akaike information criterion (AIC) did not substantially improve for any of them, we used a linear approach for all continuous variables in the final model. We did not include random slopes in our model as the focus was the contribution of variables to explaining variance, not differences in effect size between units, and random slopes were unlikely to affect the overall results of our model. Average marginal odds ratios (AMOR) were estimated using R package marginaleffects^3^.

*Multiple imputation*

For sensitivity analysis missing data was imputed by multiple imputation with chained equations respecting the nested structure of the data using R packages mice ^4^ with extension micemd^5^ and lme4^2^.

*Graphs*

Graphs were visualized using ggplot2^6^ with extensions ggpubr^7^, ggpmisc^8^ and RColorBrewer^9^.


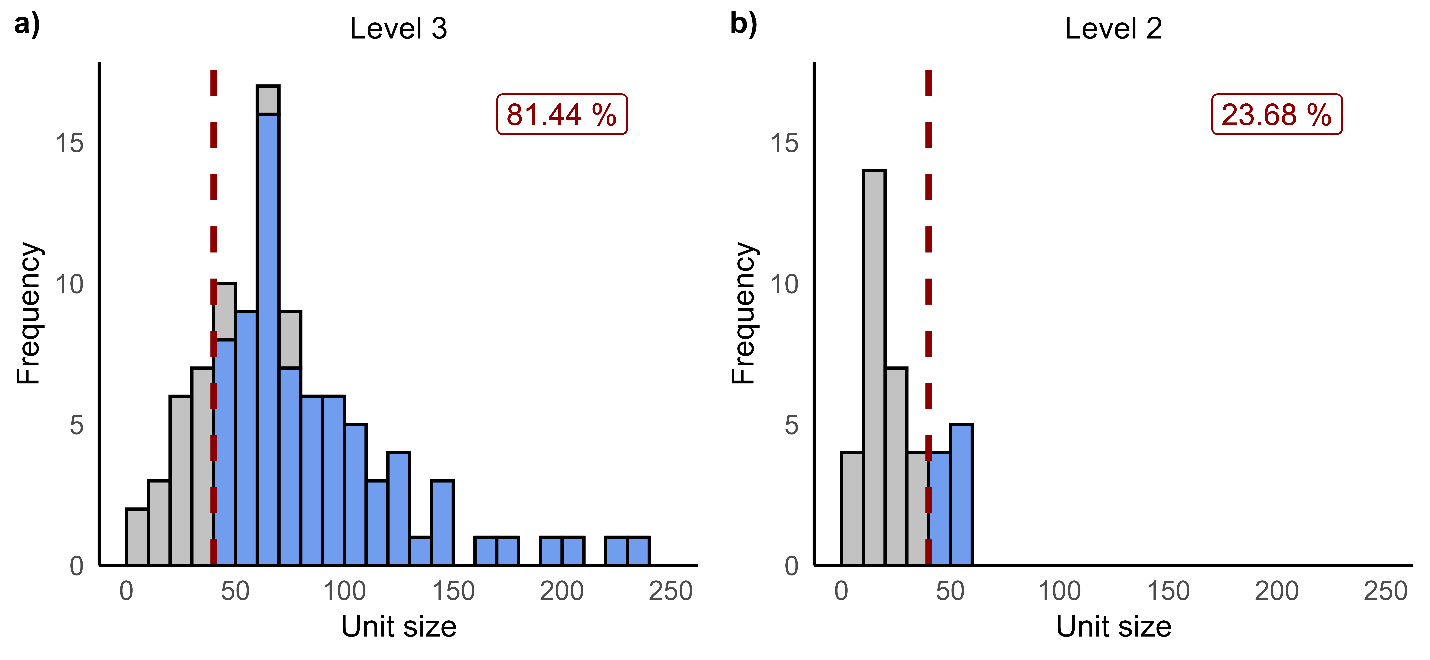


**e-Figure 1.** Histograms of the number of annual very preterm (VPT) admissions by unit for most specialised units (level 3) **(a)** and units with intermediate specialisation (level 2) **(b)**. Number of annual VPT admissions (“unit size”) was calculated by number of VPT admissions within the study period, multiplied by 2 for French units (as inclusion was only over a period of 6 months). Red line = cut-off of at least 40 annual admissions, number in red = proportion of units with at least 40 annual admissions. Blue = units included in the study, grey = units not included in the study. Discrepancy between number of included units and cut off arise from: exclusion of four French units with <40 VPT admissions within the study period but ≥ 40 annual admissions and one Estonian unit which was excluded for not providing mechanical ventilation on site.


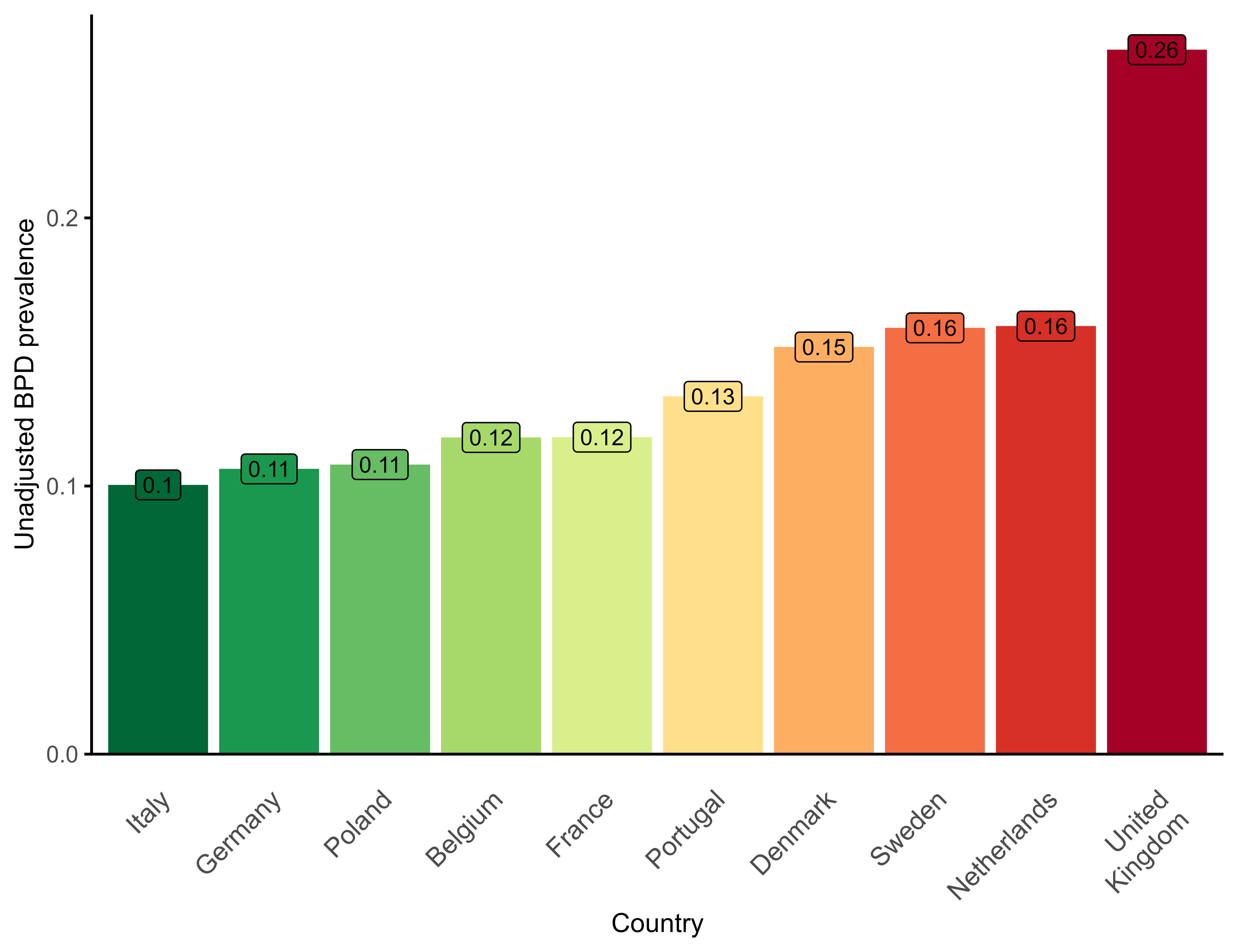


e-Figure 2. Unadjusted prevalence of bronchopulmonary dysplasia (BPD) by country.

**
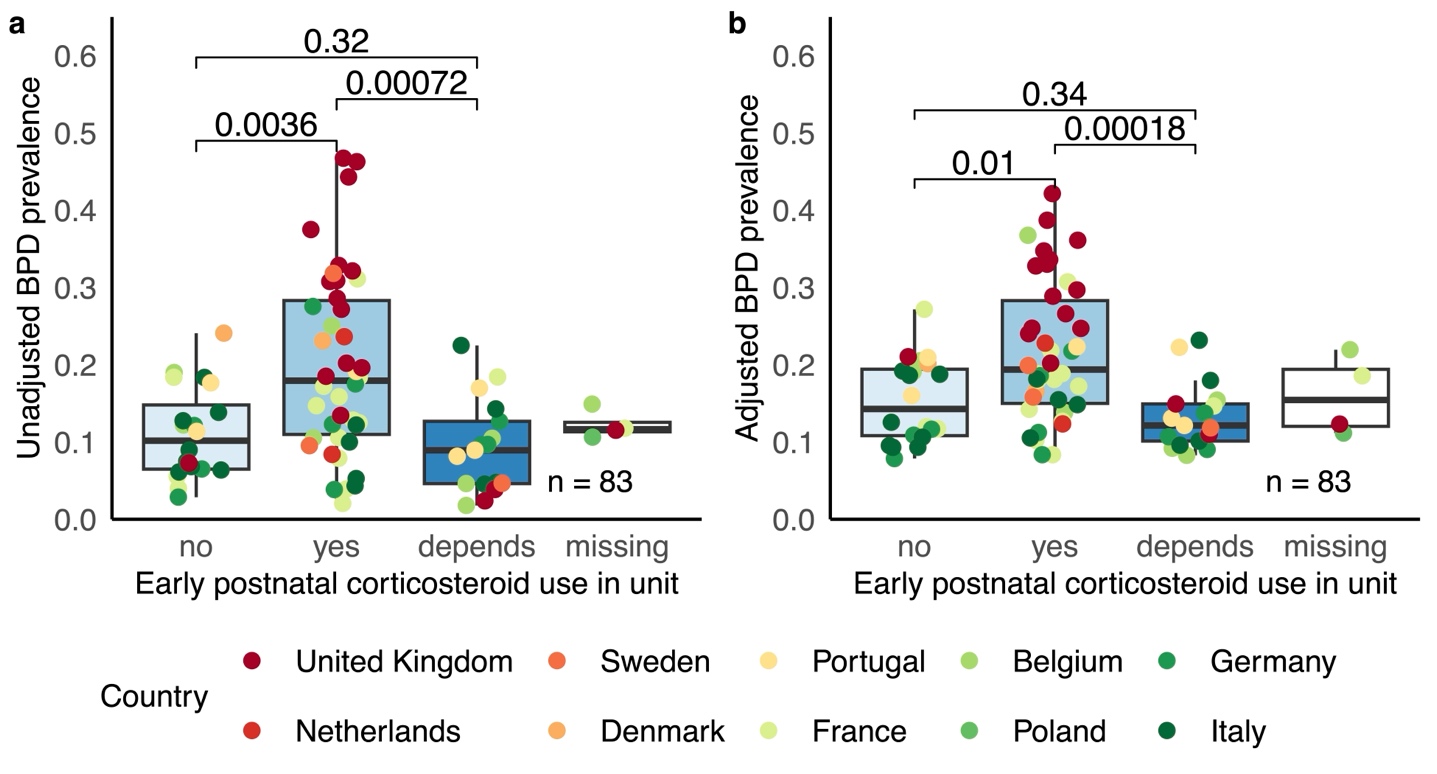
**

**e-Figure 3.** Ecological analysis of postnatal corticosteroid use. Correlation of unit’s declared use of postnatal corticosteroids for treatment of arterial hypotension or bronchopulmonary dysplasia (BPD) prevention with BPD prevalence, using the original variable with 3 levels (“yes”, “no”, “depends”). Further description of the corresponding question and factor levels can be found in e-Appendix 2.


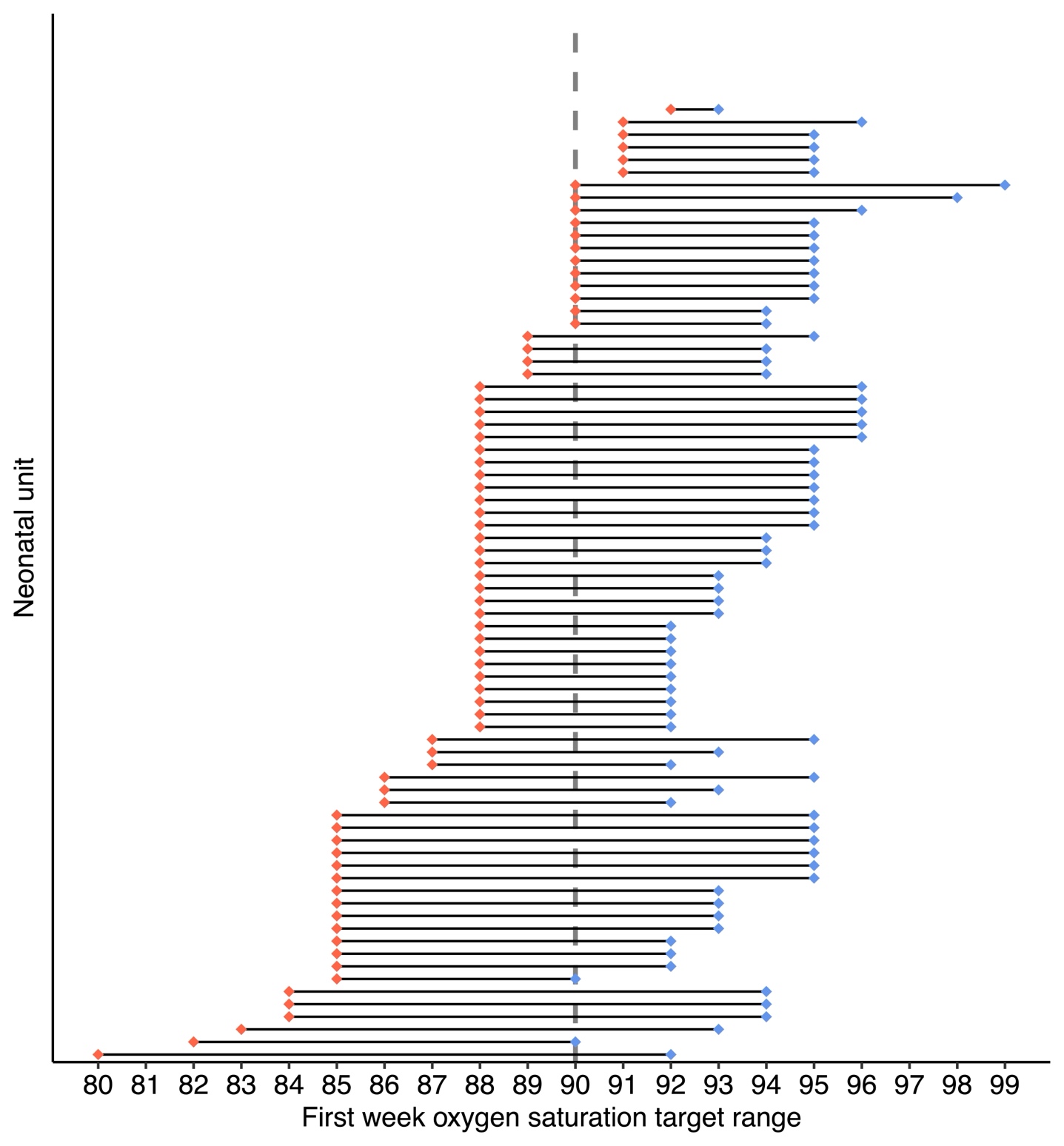


e-Figure 4. Units’ oxygen saturation target ranges in the first week of life. Showing corresponding minimum (red) and maximum (blue) oxygen saturation targets of each included neonatal unit. Demonstrating that most units could not be classified into the NeOProM target ranges of 85-89% and 90-95% ^10^.

e-Table 1. Sensitivity analysis including only infants born with a gestational age < 28 weeks compared to all included infants. Estimates are average marginal odds ratios with 95%-confidence intervals.

|  | Average Marginal Odds Ratios | |
| --- | --- | --- |
|  | < 32 weeks | <28 weeks |
| patients | n = 4,034 | n = 1,063 |
| units | k = 74 | k = 74 |
| Gestational age [weeks] | **0.49 [0.46,0.52]** | **0.54 [0.47,0.62]** |
| Birth weight z-score | **0.61 [0.56,0.66]** | **0.67 [0.59,0.75]** |
| Female sex | **0.65 [0.53,0.80]** | **0.62 [0.46,0.83]** |
| 5-minute Apgar < 7 | **1.89 [1.47,2.44]** | **1.65 [1.19,2.28]** |
| Maternal age [years] | 1.10 [0.92,1.33] | 1.07 [0.82,1.40] |
| Mother born outside Europe | **0.49 [0.37,0.66]** | 0.70 [0.48,1.01] |
| Multiparous | 0.99 [0.79,1.23] | 0.98 [0.72,1.33] |
| Admission for preterm labour | **0.73 [0.58,0.91]** | **0.72 [0.53,0.99]** |
| Antenatal corticosteroids (any) | 0.80 [0.55,1.18] | 1.03 [0.62,1.72] |
| Outborn status | 1.34 [0.92,1.94] | 1.45 [0.85,2.49] |
|  |  |  |
| Mortality [%] | 1.00 [0.97,1.03] | 0.98 [0.94,1.03] |
| Saturation targets in the first week of life [%] | **1.15 [1.06,1.25]** | **1.10 [1.00,1.21]** |
| Mechanical ventilation within 24 hours [10%] | 1.03 [0.92,1.17] | 0.96 [0.83,1.12] |
| Early postnatal corticosteroid use | **2.04 [1.35,3.09]** | **2.43 [1.46,4.06]** |
| Total annual admissions | 0.98 [0.92,1.04] | 1.03 [0.95,1.12] |

e-Table 2. Sensitivity analysis with multiple imputation of missing data on the individual level by chained equations (MICE) compared to complete case analysis (CCA). Estimates are average marginal odds ratios with 95%-confidence intervals.

|  | Average Marginal Odds Ratios | |
| --- | --- | --- |
|  | CCA | MICE |
| patients | n = 4,034 | n = 4,553 |
| units | k = 74 | k = 74 |
| Gestational age [weeks] | **0.49 [0.46,0.52]** | **0.49 [0.46,0.52]** |
| Birth weight z-score | **0.61 [0.56,0.66]** | **0.61 [0.57,0.66]** |
| Female sex | **0.65 [0.53,0.80]** | **0.60 [0.49,0.73]** |
| 5-minute Apgar < 7 | **1.89 [1.47,2.44]** | **1.83 [1.43,2.34]** |
| Maternal age [years] | 1.10 [0.92,1.33] | 1.07 [0.90,1.28] |
| Mother born outside Europe | **0.49 [0.37,0.66]** | **0.52 [0.39,0.69]** |
| Multiparous | 0.99 [0.79,1.23] | 0.99 [0.81,1.22] |
| Admission for preterm labour | **0.73 [0.58,0.91]** | **0.74 [0.59,0.91]** |
| Antenatal corticosteroids (any) | 0.80 [0.55,1.18] | 0.86 [0.60,1.22] |
| Outborn status | 1.34 [0.92,1.94] | 1.36 [0.97,1.91] |
|  |  |  |
| Mortality [%] | 1.00 [0.97,1.03] | 1.00 [0.97,1.04] |
| Saturation targets in the first week of life [%] | **1.15 [1.06,1.25]** | **1.13 [1.04,1.23]** |
| Mechanical ventilation within 24 hours [10%] | 1.03 [0.92,1.17] | 1.05 [0.93,1.19] |
| Early postnatal corticosteroid use | **2.04 [1.35,3.09]** | **1.94 [1.28,2.92]** |
| Total annual admissions | 0.98 [0.92,1.04] | 1.00 [0.94,1.06] |

**e-Table 3.** Sensitivity analysis reducing the threshold for unit inclusion from a minimum number of 40 admissions in the study period to 30. Estimates are average marginal odds ratios with 95%-confidence intervals.

|  | Average Marginal Odds Ratios | |
| --- | --- | --- |
|  | 40 admissions | 30 admissions |
| patients | n = 4,034 | n = 4,409 |
| units | k = 74 | k = 87 |
| Gestational age [weeks] | **0.49 [0.46,0.52]** | **0.49 [0.46,0.52]** |
| Birth weight z-score | **0.61 [0.56,0.66]** | **0.61 [0.56,0.66]** |
| Female sex | **0.65 [0.53,0.80]** | **0.65 [0.53,0.79]** |
| 5-minute Apgar < 7 | **1.89 [1.47,2.44]** | **1.91 [1.49,2.45]** |
| Maternal age [years] | 1.10 [0.92,1.33] | 1.08 [0.90,1.29] |
| Mother born outside Europe | **0.49 [0.37,0.66]** | **0.51 [0.38,0.68]** |
| Multiparous | 0.99 [0.79,1.23] | 1.00 [0.81,1.24] |
| Admission for preterm labour | **0.73 [0.58,0.91]** | **0.75 [0.60,0.93]** |
| Antenatal corticosteroids (any) | 0.80 [0.55,1.18] | 0.79 [0.55,1.14] |
| Outborn status | 1.34 [0.92,1.94] | 1.33 [0.93,1.92] |
|  |  |  |
| Mortality [%] | 1.00 [0.97,1.03] | 1.00 [0.97,1.03] |
| Saturation targets in the first week of life [%] | **1.15 [1.06,1.25]** | **1.15 [1.06,1.24]** |
| Mechanical ventilation within 24 hours [10%] | 1.03 [0.92,1.17] | 1.07 [0.96,1.19] |
| Early postnatal corticosteroid use | **2.04 [1.35,3.09]** | **1.94 [1.33,2.81]** |
| Total annual admissions | 0.98 [0.92,1.04] | 0.98 [0.93,1.04] |

**e-Table 4.** Sensitivity analysis using the variable “unit’s use of postnatal corticosteroids for arterial hypotension or BPD prevention” with the original three categories instead of dichotomous categorization. Estimates are average marginal odds ratios with 95%-confidence intervals. Abbreviations: ref = reference.

|  | Average Marginal Odds Ratios | |
| --- | --- | --- |
|  | 2 levels | 3 levels |
| patients | n = 4,034 | n = 4,034 |
| units | k = 74 | k = 74 |
| Gestational age [weeks] | **0.49 [0.46,0.52]** | **0.49 [0.46,0.52]** |
| Birth weight z-score | **0.61 [0.56,0.66]** | **0.61 [0.56,0.66]** |
| Female sex | **0.65 [0.53,0.80]** | **0.65 [0.53,0.80]** |
| 5-minute Apgar < 7 | **1.89 [1.47,2.44]** | **1.89 [1.46,2.43]** |
| Maternal age [years] | 1.10 [0.92,1.33] | 1.11 [0.92,1.34] |
| Mother born outside Europe | **0.49 [0.37,0.66]** | **0.49 [0.37,0.66]** |
| Multiparous | 0.99 [0.79,1.23] | 0.99 [0.80,1.23] |
| Admission for preterm labour | **0.73 [0.58,0.91]** | **0.73 [0.58,0.92]** |
| Antenatal corticosteroids (any) | 0.80 [0.55,1.18] | 0.81 [0.55,1.18] |
| Outborn status | 1.34 [0.92,1.94] | 1.33 [0.92,1.93] |
|  |  |  |
| Mortality [%] | 1.00 [0.97,1.03] | 1.00 [0.97,1.03] |
| Saturation targets in the first week of life [%] | **1.15 [1.06,1.25]** | **1.16 [1.07,1.26]** |
| Mechanical ventilation within 24 hours [10%] | 1.03 [0.92,1.17] | 1.03 [0.92,1.16] |
| Early postnatal corticosteroid use “yes” (ref: “no/depends”) | **2.04 [1.35,3.09]** |  |
| Early postnatal corticosteroid use “depends” (ref: “no”) |  | 0.61 [0.36,1.05] |
| Early postnatal corticosteroid use “yes” (ref: “no”) |  | **1.63 [1.02,2.61]** |
| Total annual admissions | 0.98 [0.92,1.04] | 0.98 [0.92,1.04] |

**e-Table 5.** Sensitivity analysis using the combined outcome of bronchopulmonary dysplasia or death instead of bronchopulmonary dysplasia (BPD). Estimates are average marginal odds ratios with 95%-confidence intervals. While infants who died before 36 weeks’ postmenstrual age stay were excluded for the BPD analysis (as BPD outcome is not available), they were included in the analysis of the combined outcome.

|  | Average Marginal Odds Ratios | |
| --- | --- | --- |
|  | BPD | BPD or death |
| patients | n = 4,034 | n = 4,456 |
| units | k = 74 | k = 74 |
| Gestational age [weeks] | **0.49 [0.46,0.52]** | **0.49 [0.46,0.51]** |
| Birth weight z-score | **0.61 [0.56,0.66]** | **0.69 [0.64,0.73]** |
| Female sex | **0.65 [0.53,0.80]** | **0.67 [0.56,0.80]** |
| 5-minute Apgar < 7 | **1.89 [1.47,2.44]** | **2.33 [1.88,2.88]** |
| Maternal age [years] | 1.10 [0.92,1.33] | 0.99 [0.84,1.16] |
| Mother born outside Europe | **0.49 [0.37,0.66]** | **0.59 [0.46,0.75]** |
| Multiparous | 0.99 [0.79,1.23] | 1.17 [0.97,1.40] |
| Admission for preterm labour | **0.73 [0.58,0.91]** | **0.70 [0.58,0.86]** |
| Antenatal corticosteroids (any) | 0.80 [0.55,1.18] | 0.77 [0.57,1.06] |
| Outborn status | 1.34 [0.92,1.94] | 1.29 [0.94,1.78] |
|  |  |  |
| Mortality [%] | 1.00 [0.97,1.03] |  |
| Saturation targets in the first week of life [%] | **1.15 [1.06,1.25]** | **1.13 [1.05,1.20]** |
| Mechanical ventilation within 24 hours [10%] | 1.03 [0.92,1.17] | 1.04 [0.94,1.14] |
| Early postnatal corticosteroid use | **2.04 [1.35,3.09]** | **1.58 [1.13,2.22]** |
| Total annual admissions | 0.98 [0.92,1.04] | 0.96 [0.91,1.01] |

**References**

1. Ash AS et al. Committee of Presidents of Statistical Societies (COPSS). Statistical Issues in Assessing Hospital Performance. Published online 2012.

2. Bates D, Mächler M, Bolker B, Walker S. Fitting Linear Mixed-Effects Models Using lme4. *Journal of Statistical Software*. 2015;67:1-48. doi:10.18637/jss.v067.i01

3. Arel-Bundock V, Greifer N, Heiss A. How to Interpret Statistical Models Using marginaleffects for R and Python. *Journal of Statistical Software*. 2024;111:1-32. doi:10.18637/jss.v111.i09

4. Buuren S van, Groothuis-Oudshoorn K. mice: Multivariate Imputation by Chained Equations in R. *Journal of Statistical Software*. 2011;45:1-67. doi:10.18637/jss.v045.i03

5. Audigier V, Resche-Rigon M, Munoz Avila J. micemd: Multiple Imputation by Chained Equations with Multilevel Data. Published online 2022. Accessed May 13, 2025. https://cran.r-project.org/web/packages/micemd/index.html

6. Wickham H. *Ggplot2: Elegant Graphics for Data Analysis*. Second edition. Springer; 2016. doi:10.1007/978-3-319-24277-4

7. Kassambara A. ggpubr: “ggplot2” Based Publication Ready Plots. Published online February 10, 2023. Accessed May 12, 2025. https://cran.r-project.org/web/packages/ggpubr/index.html

8. Aphalo PJ, Slowikowski K, Mouksassi S. ggpmisc: Miscellaneous Extensions to “ggplot2.” Published online November 14, 2024. Accessed May 12, 2025. https://cran.ma.imperial.ac.uk/web/packages/ggpmisc/index.html

9. Neuwirth E. RColorBrewer: ColorBrewer Palettes. Published online April 3, 2022. Accessed May 12, 2025. https://cran.r-project.org/web/packages/RColorBrewer/index.html

10.Askie LM, Darlow BA, Finer N, et al. Association Between Oxygen Saturation Targeting and Death or Disability in Extremely Preterm Infants in the Neonatal Oxygenation Prospective Meta-analysis Collaboration. *JAMA*. 2018;319(21):2190-2201. doi:10.1001/jama.2018.5725
